# Factors related to the frequency of citation of epidemiologic publications

**DOI:** 10.1186/1742-5573-5-3

**Published:** 2008-02-26

**Authors:** Kristian B Filion, I Barry Pless

**Affiliations:** 1 Department of Epidemiology and Biostatistics and Occupational Health, McGill University, Montreal, Quebec, Canada

## Abstract

**Background:**

Previous studies have demonstrated that the frequency with which a publication is cited varies greatly. Our objective was to determine whether author, country, journal, or topic were associated with the number of times an epidemiological publication is cited.

**Methods:**

We used outcome-based sampling and investigated one public health issue – child injury prevention, and one clinical topic – coronary artery disease (CAD) prevention. Using the Institute for Scientific Information's (ISI) Web of Science^® ^databases, we limited searches to full articles involving humans published in English between 1998 and 2004. We calculated the citation rate and, after frequency-matching on year of publication, selected the 36 most frequently cited and 36 least frequently cited articles per year, for a total of 252 highly-cited and 252 infrequently-cited articles per topic area (child injury prevention and CAD prevention).

**Results:**

Highly-cited articles in both CAD and child injury prevention were more likely to be published in medium or high impact journals or in journals with medium or high circulations. They were also more likely to be published by authors from U.S. institutions. Among articles examining CAD prevention, the highly-cited articles often involved risk factors, and the association between topics and frequency of citation persisted after adjusting for impact factor. Among articles addressing child injury prevention, topic was not statistically associated with citation.

**Conclusion:**

Journal and country appear to be the factors most strongly associated with frequency of citation. In particular, highly-cited articles are predominantly published in high-impact, high-circulation journals. The factors, however, differ somewhat depending on the area of research the journals represent. Among CAD prevention articles, for example, topic is also an important predictor of citation whereas the same is not true for articles addressing injury prevention.

**Condensed Abstract:**

Our objective was to determine whether author, country, journal, or topic were associated with the number of times an epidemiological publication is cited. We used outcome-based sampling and investigated one public health issue, child injury prevention, and one clinical topic, coronary artery disease (CAD) prevention. Using the Institute for Scientific Information (ISI) Web of Science^® ^databases, we limited searches to full articles involving humans published in English between 1998 and 2004. We calculated the citation rate and, after frequency-matching on year of publication, selected the 36 most frequently cited and 36 least frequently cited articles per year, for a total of 252 highly-cited and 252 infrequently-cited articles per topic area (child injury prevention and CAD prevention). Highly-cited articles in both CAD and child injury prevention were more likely to be published in medium or high impact journals or in journals with medium or high circulations. They were also more likely to be published by authors from U.S. institutions. Among articles examining CAD prevention, the highly-cited articles often involved risk factors, and the association between topics and frequency of citation persisted after adjusting for impact factor. Among articles addressing child injury prevention, topic was not statistically associated with citation.

## Introduction

The mission of this Journal includes publishing "epidemiology-based policy analysis, critical analyses of the field and its practices, or various contributions to methodology, philosophy, or other perspectives on the field" [1]. As is true for most journals, *Epidemiologic Perspectives and Innovations *wishes to publish work that is influential at many levels, including clinical practice, health policy, and future research. One objective measure of the influence of a publication on future research is the frequency with which the study is cited in subsequent publications. "Citationology", a term coined by Garfield, refers to "the theory and practice of citation, including its derivative disciplines citation analysis and bibiometrics" [2]. Citationology has been examined in many fields, ranging from sociology [3] to health sciences [4]. In these studies, much attention has been focused on the large body of literature that is uncited and somewhat less on the characteristics of the relatively few journals in which a large proportion of all cited work is published [5].

In a two-article series using data from the Institute for Scientific Information (ISI), Hamilton found that approximately 46% of papers in medicine remain uncited 5 years after publication [6,7]. However, as Pendlebury noted in a letter to the editor, this may be an overestimate because Hamilton's statistics include every type of publication, including obituaries, editorials, abstracts, letters, and other marginalia which, together, represent about 27% of all items indexed [8].

In another study, Callaham et al. investigated journal prestige and other characteristics associated with the citation of studies in peer-reviewed journals and found that the impact factor was the most important predictor [9]. Factors related to citation frequency differed depending on the field of enquiry, however.

In this study, we sought to determine if author, institution, country, journal, or topic are associated with the number of times a publication is cited. To test the robustness of our findings we chose to examine these relationships in 2 highly contrasting areas, child injury prevention and coronary artery disease (CAD) prevention, both of which were viewed from an epidemiological perspective.

## Materials and methods

We used outcome-based sampling to examine this relationship and searched the ISI Web of Science^®^, including the Science Citation Index Expanded™ (SCI EXPANDED™), the Social Sciences Citation Index (SSCI), and the Arts & Humanities Citation Index (A&HCI). For child injury prevention, we used the search terms "child*" and ("injury" or "accident") and "prevention", and for CAD prevention, we used ("coronary artery disease" or "coronary heart disease") and "prevention". We performed this search in March 2007. All searches were limited to full articles containing original research involving human subjects and to articles published in English between 1998 and 2004. We examined the results year by year. To minimize the potential effects of different latency periods between publication of the original manuscript and publication of the citing manuscript, we limited the search to articles published between 1998 and 2004, thus providing at least 2 years for the most recent citing manuscript to be published.

We assumed that the number of citations is affected by the time since publication, e.g., that an article published in 1998 is likely to have been cited more frequently than one published in 2004. To account for this, we calculated the citation rate using the following formula:

$$\text{Citation rate}=\frac{\text{Number of Citations as of March 2007}}{\text{(2005 }-\text{Year of Publication)}}$$

Consequently, if an article published in 2000 was cited 300 times, its citation rate would be 300/(2005-2000) = 60 citations per year. To minimize bias, we also used frequency-matching, selecting the 36 most frequently cited and 36 most infrequently cited articles for each year for each topic area (child injury prevention and CAD prevention). With 36 highly-cited and 36 infrequently cited articles per topic per year, our final sample had 252 highly-cited and 252 infrequently-cited articles examining child injury prevention and 252 highly-cited and 252 infrequently-cited articles examining CAD prevention.

For each article we recorded the following characteristics: authors, journal name and type, topic, institution, country, and year of publication. In addition, we obtained the circulation for each journal from Ulrich's International Periodicals Directory. Because previous circulation data were not available, we used current circulation as a proxy, and categorized the results as low (<18,000), medium (18,000 – 50,000), or high (>50,000) circulation. Impact factors for 2001 were obtained from ISI Web of Knowledge. A journal's impact factor represents the mean number of times an article published in the last two years was cited [10]. We categorized impact factors as low (<2.7), medium (2.7 – 9.0), or high (≥10.0). These 2001 impact factors were used as a proxy for the average of each journal's impact factors over the study period.

Among publications examining child injury prevention, we classified articles according to the following topics: burns/fire, education, guns, motor vehicle, playground/sporting, or other injuries. Among those examining CAD prevention, we grouped topics into medications, new technologies, risk factors, or other issues.

Statistical analyses consisted first of a descriptive phase where we provided the distribution of the following characteristics: authors, institution, country, topic, and journals by name, type, circulation, and impact factor. Nominal data are presented as counts or proportions, and continuous data as means ± standard deviation (SD). In a subsequent analytic phase, Fisher null hypothesis tests were used to examine the association between whether a publication is highly or infrequently cited and the following factors: country, topic, and journal name, type, circulation, and impact factor. Nominal data were compared using Chi-square or Fisher's exact tests, as appropriate, and continuous data using student t-tests. We also used the stratified (Cochran-) Mantel-Haenszel to test the association across impact factor strata. All statistical analyses were performed using SAS 9.1 (SAS, SAS Institute, Gary, NC).

## Results

### Author, institution, and country

The CAD prevention articles involved over 2,500 authors whereas the child injury prevention articles involved 1,700 authors. Over 90% of authors appeared on only 1 publication. Most institutions of origin also only appeared on only 1 publication during the study period. Institutions with more than 2 publications are listed in Tables 1 and 2. The Centers for Disease Control had the greatest number of highly-cited publications on injury prevention, whereas Harvard University produced the most highly-cited research papers on CAD prevention.

**Table 1:** Institutions of highly-cited and infrequently-cited publications examining coronary artery disease prevention (%).

Institution	Highly-Cited	Infrequently-Cited
Harvard University	11.9	0
National Heart Lung and Blood Institute (USA)	2.8	0
National Public Health Institute (Australia)	2.0	0
University of California at San Francisco	2.0	0
University of Texas	2.0	2.0
Cleveland Clinic	1.6	0
Centre for Disease Control and Prevention	1.6	0
University of Munster	1.6	0
Baylor College of Medicine	1.2	1.6
National Heart and Lung Institute (UK)	1.2	0
Tufts University	1.2	0
University of Glasgow	1.2	1.6
University of Massachusetts	1.2	0
University of Southern California	1.2	2.0
University of Washington	1.2	0
University of Birmingham	0	1.2
University of Chicago	0	1.2
University of London, Imperial College of Science, Technology, and Medicine	0	1.2
University of North Carolina	0	1.2
University of Ottawa	0	2.0
University of Toronto	0	1.2
		
Other*	66.1	84.8

* Includes all institutions with 2 or fewer publications included in our study

**Table 2:** Institutions of highly-cited and infrequently-cited publications examining child injury prevention (%).

Institution	Highly-Cited	Infrequently-Cited
Centre for Disease Control and Prevention	5.2	0.4
University of Guelph	2.8	0
American Academy of Pediatrics	2.4	0
University of Alabama	2.4	0
Johns Hopkins	2.0	0
Children's Hospital (Philadelphia)	1.6	0
National Institute of Child Health and Human Development	1.6	0
University of Pittsburgh	1.6	0
Children's Hospital (Pittsburgh)	1.2	2.0
Ohio State University	1.2	0
Pacific Institute for Research and Evaluation	1.2	0
Queens University	1.2	0
University of Iowa	1.2	0
University of Ottawa	1.2	0
University of Sao Paulo	1.2	0
Hospital for Sick Children	0	1.2
Karolinska Institutet	0	1.6
Tel Aviv University	0	1.2
University of Athens	0	1.2
University of Auckland	0	1.6
University of California at Los Angeles	0	1.2
University of Missouri	0	1.2
University of North Carolina	0	1.2
University of New Mexico	0	1.2
		
Other*	72.0	86.0

* Includes all institutions with 2 or fewer publications included in our study

Studies conducted in the United States were more likely to be highly-cited than those conducted in other countries. This relationship was statistically significant for both topic areas (Tables 3 and 4).

**Table 3:** Characteristics of highly-cited and infrequently-cited publications examining coronary artery disease prevention

Characteristic	Highly-Cited	Infrequently-Cited	P-Value
Year (mean ± SD)	2000.9 ± 2.0	2000.9 ± 2.1	0.66
Journal Name (%)			
Circulation	23.4	0.8	<0.0001
Lancet	6.8	0	
JAMA	12.3	0	
Journal of the American College of Cardiology	4.0	0	
New England Journal of Medicine	7.9	0	
Other	45.6	99.2	
Journal Type (%)			
Cardiovascular	35.2	27.8	<0.0001
Endocrinology/Metabolism	3.9	5.6	
General Medicine	43.4	20.6	
Health Services or Heath Policy	0	7.1	
Nutrition/Diet	4.7	4.0	
Pharmacology	2.3	9.9	
Public, Environmental, or Occupational Health	3.1	7.5	
Other	7.4	17.5	
Journal Circulation (%)			
High	36.1	6.0	<0.0001
Medium	42.1	7.5	
Low	21.8	86.5	
Journal Impact Factor (%)			
High	54.4	1.6	<0.0001
Medium	33.7	10.7	
Low	11.9	87.7	
Topic (%)			
Medications	44.5	42.5	0.04
New Technologies	4.0	3.6	
Risk Factors	36.9	29.8	
Other	14.7	24.2	
Country (%)			
United States	61.5	32.9	<0.0001
United Kingdom	16.7	11.9	
Other	21.8	55.2	

Abbreviations: SD = standard deviation, JAMA = Journal of the American Medical Association

**Table 4:** Characteristics of highly-cited and infrequently-cited publications examining injury prevention among children.

Characteristic	Highly-Cited	Infrequently-Cited	P-Value
Year (mean ± SD)	2000.9 ± 2.0	2000.9 ± 2.1	0.66
Journal Name (%)			
Archives of Pediatric and Adolescent Medicine	3.6	0.8	<0.0001
Burns	4.0	3.2	
Injury Prevention	4.0	4.0	
Journal of Burn Care and Rehabilitation	0.4	4.0	
Journal of Paediatric and Child Health	1.2	3.6	
Journal of Paediatric Surgery	2.8	2.8	
Journal of Trauma	3.4	1.6	
Paediatrics	19.4	4.8	
Other	61.1	75.4	
Journal Type (%)			
Critical Care/Emergency Room	11.8	16.3	0.12
Education	2.0	3.2	
General Medicine	9.1	10.3	
Other	20.1	23.4	
Paediatric	30.7	21.0	
Psychology	10.2	6.7	
Public, Environmental, or Occupational Health	16.1	19.0	
Journal Circulation (%)			
High	27.4	5.2	<0.0001
Medium	10.7	5.2	
Low	61.9	89.7	
Journal Impact Factor (%)			
High	4.0	0.0	<0.0001
Medium	29.0	5.2	
Low	67.1	94.8	
Topic (%)			
Burns/Fire	6.4	10.3	0.42
Education	8.3	8.3	
Guns	6.0	4.0	
Motor Vehicle Accidents	7.9	8.7	
Playgrounds/Sports	13.1	9.5	
Other	59.1	58.3	
Country (%)			
United States	60.7	43.7	0.0002
United Kingdom	11.1	11.5	
Other	28.2	44.8	

Abbreviations: SD = standard deviation

### Journal

Highly-cited papers were most likely to be published in journals with large circulation or high impact factors, and circulation and impact factor were highly correlated (r = 0.77). Thus, among CAD prevention publications, highly-cited articles were more likely to be published in Circulation, the Journal of the American College of Cardiology, the Journal of the American Medical Association, the Lancet, or the New England Journal of Medicine rather than other journals (Table 3).

For injury prevention papers, highly-cited publications appeared in the Archives of Pediatric and Adolescent Medicine, Burns, Injury Prevention, the Journal of Trauma, and Pediatrics (Table 4). Thus, in both categories, highly-cited publications appeared in journals with medium or high circulation and medium or high impact factors (Tables 3, 4) and infrequently-cited papers were in journals with low impact factors or low circulation.

### Topic

Topic was also associated with citation frequency only among CAD prevention papers. Highly-cited articles were more likely to discuss risk factors, whereas infrequently cited papers addressed other, general topics (Table 3). This association persisted after adjusting for impact factor (p = 0.04).

For child injury prevention, there was no significant association between topic and frequency of citation (Table 4). Infrequently-cited papers may be more likely to examine burn prevention than highly-cited ones, but this relationship did not reach statistical significance and remains after adjusting for impact factor (p = 0.68).

## Discussion

Perhaps not surprisingly, the journal, reflecting in part impact factor and circulation, was most strongly associated with the frequency with which a publication was cited. This was particularly evident among publications examining CAD prevention, where almost 80% of highly-cited articles were in medium- or high-impact journals compared with 12% of infrequently-cited publications. For CAD, we also found topic to be associated with frequency of citation and to the country where the research was done. For both CAD and injury prevention they were far more likely to come from the United States.

The factors found to be related to frequency of citation are themselves highly correlated, and these correlations must be considered when interpreting these results. For example, it is likely that journal of publication lies in the causal pathway because high-impact journals more often publish articles about interesting or timely topics. Consequently, adjusting for intermediate variables such as journal, impact factor, or circulation is likely to result in an underestimation of the effect of topic on citation frequency. In addition, impact factor is derived from how often a journal is cited. It is thus difficult to interpret the importance of the association between impact factor and future citation. Furthermore, adjusting for impact factor, which is highly correlated with frequency of citation by definition, may attenuate the observed effect of topic. However, similar results were obtained when adjusting for journal circulation instead of impact factor. This indicates the importance of choosing the 'best' journal (high circulation, high impact, or both) if authors hope to influence readers by virtue of the frequency with which their work is likely to be cited by others.

Only a small number of studies in the epidemiological literature have examined publication characteristics associated with citation frequency. The most prominent of these was performed by Callaham et al. [9]. They found impact factor to be the most important predictor, as did we. Additional important predictors were subjective newsworthiness (from a Delphi panel rating), sample size, and the presence of a control group. Perhaps surprisingly, statistically significant results were not found to be an important predictor of citation frequency.

In another study, Callaham et al. examined the citation rate among articles submitted to emergency medicine research journals compared with non-emergency medicine journals [11]. They found that the average citation rate among the emergency medicine journals was 2.04 times per year whereas the citation rate among similar articles published in non-emergency medicine journals was at least twice as high. Fifteen percent of articles published in emergency medicine journals were never cited, compared with only 5% of those published in non-emergency medicine journals. This finding could be attributable to the fact that the mean impact factor of the non-emergency medicine journals was substantially higher than that of the emergency medicine journals (4.3 vs 1.5), which further highlights the importance of impact factor as a predictor of the frequency of citation.

Previous citation studies have also examined the rates of papers that are never cited [12]. Schwartz found that 46% of all publications in medicine (including marginalia) were never cited by other authors [12]. Considering only full articles, the proportion was 22% – similar to rates from other physical sciences, including Physics (47% among all publications, 17% among articles only), Biological Sciences (41% and 19%, respectively), and Mathematics (55% and 26%, respectively). The proportion of uncitedness in these fields is substantially lower than those of other disciplines, including the Humanities (98% among all publications, 93% among articles only) and Social Sciences (75% and 48%, respectively).

The work conducted by Schwartz also highlights a key challenge in citationology – the definition of the 'population at risk'. It remains unclear whether all publications in journals catalogued by the ISI should be considered 'at risk' for citation or whether analyses should be restricted to original articles. Furthermore, given the imperfect sensitivity of literature search tools, even well-defined searches will likely produce incomplete results. As a result, it is difficult to identify the entire 'population at risk' of being cited or to calculate measures such as the risk of citation.

These difficulties in identifying the 'population at risk' also prevent the use of Kaplan-Meier survival analysis which could provide valuable information regarding time to citation – a dimension of likely relevance for many authors.

**Figure 1:**
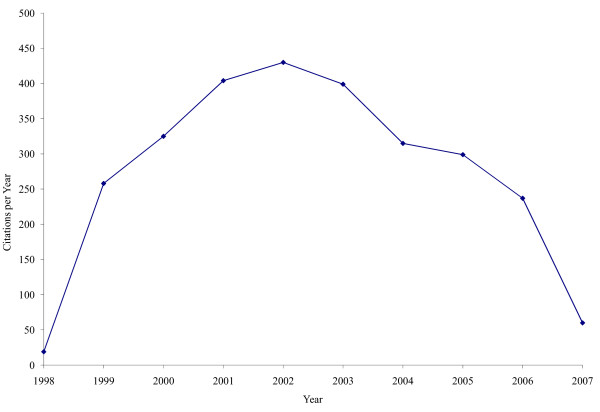
Citation trajectory of one highly-cited article examining CAD prevention. This manuscript was published in 1998, and these citation data were extracted in April 2007.

Another important challenge in examining citations is the effect of time. Calendar time can affect the citation of an article in three important ways. First, there is the potential effect of partial years. It is unlikely that an article will be published on January 1^st ^and equally unlikely that a literature search will be conducted on December 31^st^. Consequently, both the year of publication and the year of search are, in fact, partial years, the effects of which need to be considered when interpreting the results. To minimize the effects of these partial years, we frequency-matched on year of publication. Second, there is a latency period between the decision to cite an article and the publication of the citing article. This latency period can be highly variable, depending on the number of times the article is submitted, different review times, and the duration of the 'in press' period. Although the increasing popularity of open access and electronic journals likely will shorten this period, a minimum, unavoidable latency period will always remain. In the present study, therefore, we restricted our analysis to papers published between 1998 and 2004, thus allowing at least two years for the citing manuscript to be published. Third, the citation trajectory of most articles is concave-down (Figure 1); i.e., most articles experience a period of increasing citation followed by one of decreasing citation. As such, it is perhaps too simple to just examine rates of citations per year. Calendar time-citation interaction is particularly important when focusing on specific citation rates.

## Limitations

Our study has several limitations. First, to detect important differences for some variables (e.g., author, institution), even this relatively large sample is insufficient. It is also possible that the significant results may be the result of type 1 errors.

Second, although our results are generally consistent for CAD prevention and child injury prevention, the generalizability of the findings is uncertain. Additional studies involving other epidemiologic topics will be needed to determine how universal these results are.

Third, we used 2001 impact factors and assumed them to be roughly the average of any journal's impact factors over the study period. Although it would have been preferable in a design sense to have used 1998 impact factors, because the impact factors of some journals have risen sharply over the last 12 years [13], these may not be representative of later values. However, with a maximum of only 4 years between publication and the 2001 midpoint and the categorization of impact factor as low, medium, or high, it is unlikely that the use of 2001 impact factors as a proxy introduces an important bias.

Fourth, we used current circulation data as a proxy because earlier circulation data were unavailable. This is not ideal, but journal subscriptions do not greatly fluctuate over a 7 year period and it is especially unlikely that a journal would move from one circulation category (low, medium, or high) to another over this time. Also, due to the presence of institutional subscriptions, available subscription data likely underestimate the number of readers of a given journal.

Finally, as discussed previously, there are the potential effects of calendar time that we have tried to minimize by frequency-matching and by incorporating a minimum latency period into our study design.

## Conclusion

Journal, impact factor, and circulation were the factors significantly associated to the frequency of citation of epidemiologic publications. These associations were present for publications addressing child injury prevention and those examining CAD prevention. Topic also appeared to be associated with citation frequency among CAD prevention articles.

## Authors' contributions

KF contributed to study design, data collection and analysis, interpretation of results, and drafting and revising the manuscript. IBP contributed to study design, data collection, interpretation of results, and revising the manuscript.
